# Arabic web-based educational resources on iron deficiency anemia: an infodemiological study

**DOI:** 10.3389/fpubh.2025.1721461

**Published:** 2025-12-10

**Authors:** Hassan Alradhi, Hussain Aljumah, Mujtaba Alzuwayr, Ajwan Alhassan, Yaqeen Al-essa, Zaenb Alsalman, Mortadah Alsalman

**Affiliations:** 1 College of Medicine, King Faisal University, Al-Ahsa, Saudi Arabia; 2 Department of Family and Community Medicine, College of Medicine, King Faisal University, Al-Ahsa, Saudi Arabia; 3 Department of Medicine, College of Medicine, King Faisal University, Al-Ahsa, Saudi Arabia

**Keywords:** iron deficiency anemia, quality, readability, Arabic web resources, digital health literacy

## Abstract

**Introduction:**

Iron deficiency anemia (IDA) is the most widespread nutritional deficiency worldwide. This study aimed to evaluate the quality, reliability, and readability of Arabic web-based resources on IDA.

**Materials and methods:**

A retrospective, infodemiological descriptive study was conducted using validated assessment tools. A web-based search was performed on July 8, 2025, and the collected websites were evaluated using validated assessment tools, including DISCERN, JAMA, FKGL, SMOG, and FRE.

**Results:**

Of 36 included websites, most were from medical institutions or health portals. Overall reliability was limited (mean JAMA score 1.39 ± 0.96), and no website met all JAMA criteria. Website quality was generally moderate (DISCERN 39.72 ± 8.83), with governmental and public health websites performing poorly (p < 0.001). Readability was high (FKGL 3.65 ± 3.58; SMOG 3.26 ± 0.79; FRE ≥ 80 in 97.2%). JAMA and DISCERN scores were positively correlated (ρ = 0.430, *p* = 0.009).

**Conclusion:**

Arabic-language web resources on IDA are easily readable but demonstrate significant deficiencies in quality and reliability. This pronounced gap may contribute to misinformation, delayed care-seeking, and suboptimal management. Collaboration between medical institutions, public health organizations, and digital platforms will be essential for developing standardized, evidence-based patient education materials, which could support earlier intervention and help reduce the public health burden of IDA in Arabic-speaking communities.

## 1 Introduction

Iron deficiency anemia (IDA) is the most widespread nutritional deficiency worldwide. As of 2021, nearly two billion people are affected by anemia, with iron deficiency accounting for around two-thirds of these cases (1, 2). IDA is particularly common among women of reproductive age, children, and individuals living in low-income regions where access to iron-rich foods and healthcare is limited (3). In Middle Eastern countries, prevalence rates range from 12.6 to 50% among schoolchildren and from 22.7 to 54% among pregnant women (4).

The internet has become one of the most commonly used sources for health information globally. People frequently search online to learn about disease symptoms, diagnosis, dietary advice, and treatment options. However, online resources vary widely in reliability, ranging from personal opinions to scientifically validated information (5, 6). This variability complicates the ability of users to discern trustworthy sources, particularly when making health-related decisions (5). Concerns about the quality of online health information are widespread but tend to be less pronounced among English speakers. This is largely because most scientific literature is published in English, offering easier access to peer-reviewed resources. In contrast, non-English speakers often face limited access to high-quality, medically reviewed content, making the provision of health information in one's native language crucial for equity in healthcare (5, 7).

Infodemiology, a concept introduced by Eysenbach, focuses on studying the distribution and determinants of online health information and its impact on public health behaviors. It offers a framework for assessing the quality, reliability, and accessibility of digital health resources and for identifying gaps that may affect health literacy and decision-making (8).

Arabic is the sixth most spoken language in the world and the primary language across the Middle East, where IDA is notably prevalent. Despite this, to our knowledge the quality of Arabic-language websites addressing IDA has not been systematically evaluated before. With growing reliance on the internet for health-related information, it is essential to ensure that available resources are accurate, trustworthy, and easy to understand (7, 9). To address this gap, this study applies validated infodemiological tools to evaluate the quality, reliability, and readability of Arabic-language online content on IDA.

## 2 Materials and methods

### 2.1 Study design

This was a retrospective, infodemiological descriptive study evaluating Arabic-language online educational resources related to IDA. Search terms for “iron deficiency” in Arabic were derived from Google Trends data.

### 2.2 Search strategy

A web-based search was conducted on July 8, 2025, using three major search engines (Google, Yahoo, and Bing). To reflect typical user behavior, the first 100 consecutive results (10 pages) from each engine were screened. Searches were performed in incognito mode without login to minimize algorithmic bias, and all were completed within 24 h to reduce temporal variation.

**Figure 1:**
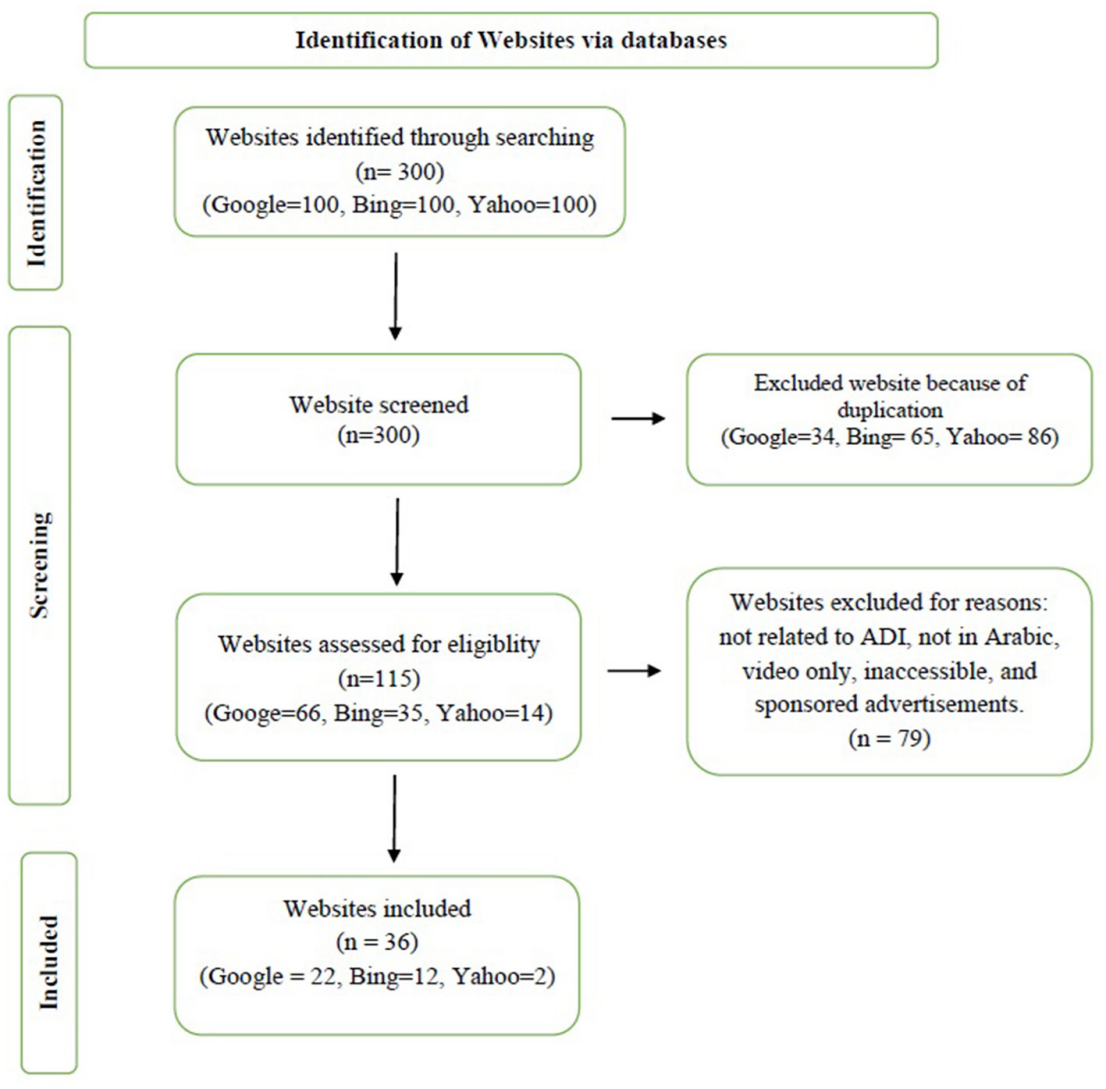
PRISMA flow diagram.

Websites were eligible if they were in Arabic, publicly accessible, and primarily intended to educate patients or the public about IDA. Exclusion criteria are outlined in Figure 1. The included websites were categorized into six typologies: medical institutions or hospitals, health portals and general educational websites, governmental websites, public health organizations, blogs, and pharmacy-affiliated sites. Each website was evaluated for coverage of IDA-related topics (definition, signs/symptoms, causes, risk factors, complications, prognosis, treatment, and prevention). Each website was independently assessed by two evaluators using the DISCERN instrument. The evaluations were conducted by two trained senior medical students, under the direct supervision of a Consultant Hematologist and a Consultant Family Physician to ensure accuracy and consistency. Inter-rater reliability was evaluated using Cohen's weighted kappa, resulting in excellent agreement (κ = 0.797), which confirms the consistency and reliability of the quality assessments.

### 2.3 Quality, reliability, and readability assessment

Websites were assessed using two validated instruments: the DISCERN tool and the Journal of the American Medical Association (JAMA) benchmarks. The DISCERN instrument consists of 16 items with total scores ranging from 16 to 80. It is divided into three sections: items 1–8 evaluate the reliability of the information, items 9–15 assess the clarity and balance in presenting treatment options, and item 16 provides an overall quality rating. Each item is scored on a five-point scale, and total scores are categorized as high quality (≥65), moderate quality (33–64), or low quality (16–32).

The JAMA benchmarks assess the trustworthiness of online health information across four criteria: (1) authorship (disclosure of authors, affiliations, and credentials), (2) attribution (presence of references and sources), (3) disclosure (transparency about ownership, funding, and conflicts of interest), and (4) currency (indication of publication or update date). Each criterion earns one point if satisfied, yielding a total score between 0 and 4. Scores of 3 or higher suggest high reliability, whereas lower scores indicate limited trustworthiness (10, 11).

Readability was assessed using an online tool that applies validated readability tools, including the Flesch–Kincaid Grade Level (FKGL), Simple Measure of Gobbledygook (SMOG), and Flesch Reading Ease (FRE). Although originally developed for English, these tools have been applied to other languages for approximate readability estimation (5, 12–15). Following recommended thresholds, acceptable readability was defined as FRE ≥ 80 and FKGL/SMOG < 7 (16, 17). The FKGL index ranges from 0 to 18, with higher scores reflecting greater reading difficulty. The SMOG index estimates the years of education required to understand a text, with scores of 7–8 corresponding to material suitable for readers at the 7th−8th grade level. FRE scores range from 0 to 100, where higher values indicate easier readability: 90–100 is considered very easy, 80–89 easy, 70–79 fairly easy, 60–69 standard (appropriate for the general public), 50–59 fairly difficult, and < 50 difficult (suitable for college or graduate-level readers). All assessments were conducted using automated formulas embedded in the tool, which has been validated in peer-reviewed literature (13–15). In addition, the evaluation followed recommendations from the American Medical Association and the U.S. Department of Health and Human Services, which advise that patient education materials be written at or below a 5th−6th grade level to maximize accessibility (16, 17).

### 2.4 Statistical analysis

All analyses were performed using SPSS version 27 (IBM Corp.). Descriptive statistics were reported as frequencies and percentages for categorical variables, and as mean, and standard deviation (SD) for continuous variables. The Shapiro–Wilk test was used to assess the normality of continuous data. Categorical variables, such as website typology and adherence to JAMA criteria, were compared using chi-square tests. Spearman's rho correlation analysis was applied to examine relationships between DISCERN and JAMA scores, as well as their associations with readability indices (FRE, FKGL, and SMOG). A *p*-value < 0.05 was considered statistically significant.

## 3 Results

A total of 300 websites were initially identified. After removing duplicates, 115 unique websites remained. Following application of exclusion criteria, 36 websites met all inclusion criteria and were included for analysis (Figure 1).

**Figure 2:**
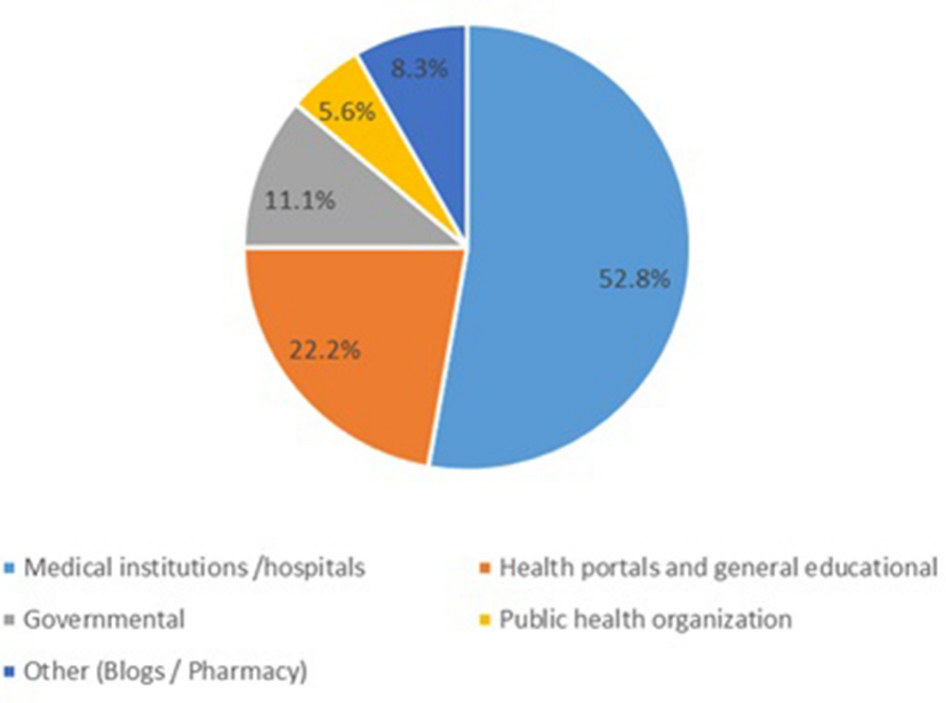
Distribution of the included websites according to website typology (*n* = 36).

**Figure 3:**
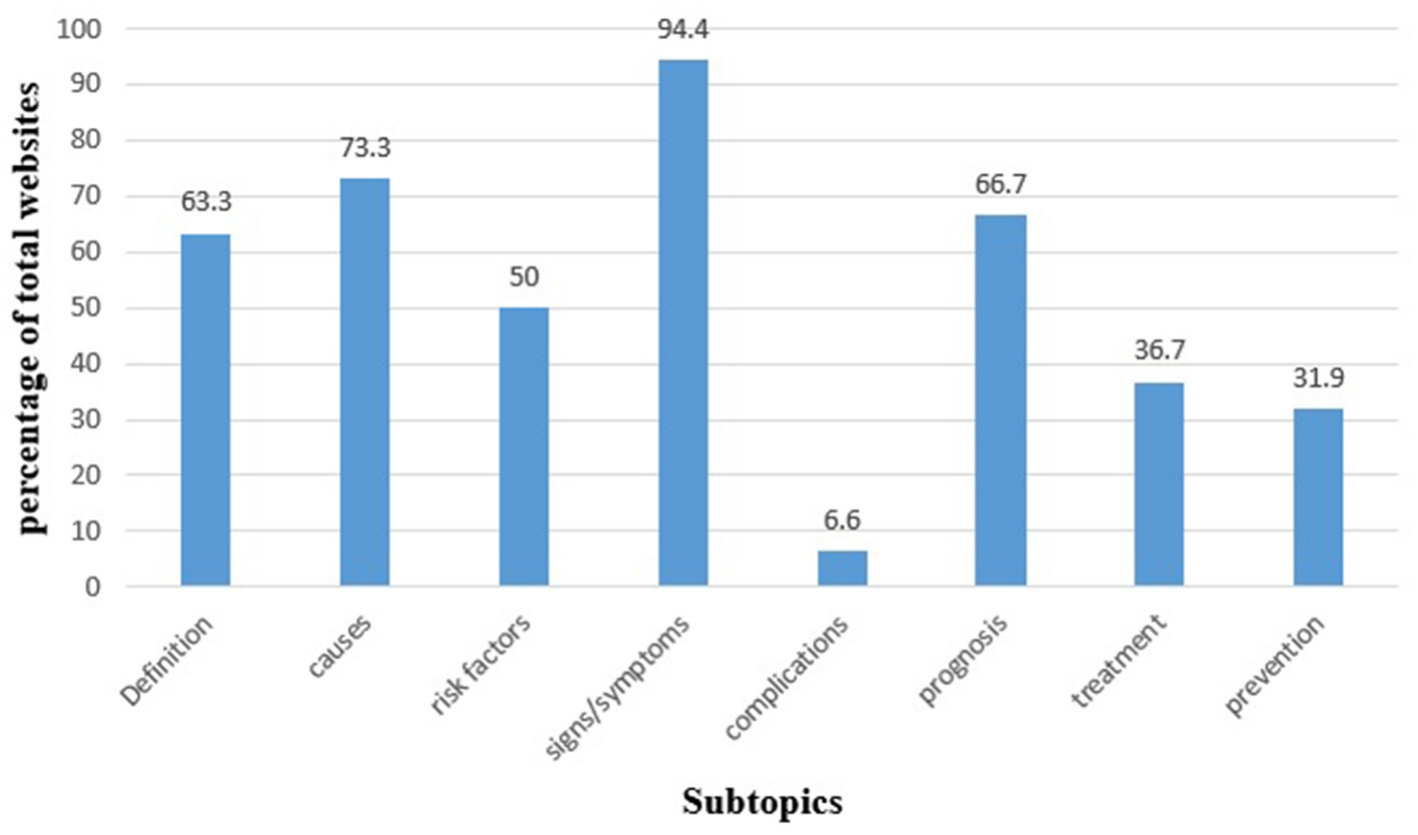
Subtopic analysis of the included websites (*n* = 36).

Regarding website typology, more than half of the included websites (52.8%) were developed by medical institutions or hospitals. Health portals and general educational websites accounted for 22.2%, followed by governmental websites at 11.1%. Public health organization websites represented 5.6%, while the remaining 8.3% were blogs or pharmacy-affiliated sites (Figure 2). Subtopic analysis revealed that the majority of websites addressed signs and symptoms, followed by causes and prognosis, whereas treatment, prevention, and complications were infrequently covered (Figure 3).

### 3.1 Website reliability, quality, and readability

Overall, the reliability of Arabic-language IDA websites was limited. According to JAMA benchmarks, the mean score was 1.39 ± 0.96, and none of the included websites fulfilled all four criteria. Thirteen websites (36.1%) satisfied only one item, and 11 (30.6%) satisfied two items. Authorship and currency were reported in 50% of websites, while disclosures were rarely provided (5.6%). Health portals showed relatively higher compliance, with 50% achieving two items and 37.5% reaching three, whereas governmental and public health organization websites consistently underperformed (Table 1).

**Table 1:** Quality of the included websites based on affiliation (*n* = 36).

**Indicator**	**Response**	**Med. Inst. & Hosp. (*n* = 19)**	**Health Portals & GEW (*n* = 8)**	**Governmental (*n* = 4)**	**Public Health Orgs. (*n* = 2)**	**Other (Blog & Pharm.) (*n* = 3)**	**Total (*n* = 36)**	***p*-value**
Number of Achieved JAMA Items per Website	0	4 (21.1%)	1 (12.5%)	1 (25.0%)	1 (50.0%)	0 (0.0%)	7 (19.4%)	0.416
	1	8 (42.1%)	0 (0.0%)	2 (50.0%)	1 (50.0%)	2 (66.7%)	13 (36.1%)	
	2	5 (26.3%)	4 (50.0%)	1 (25.0%)	0 (0.0%)	1 (33.3%)	11 (30.6%)	
	3	2 (10.5%)	3 (37.5%)	0 (0.0%)	0 (0.0%)	0 (0.0%)	5 (13.9%)	
Authorship	Present	9 (47.4%)	6 (75.0%)	1 (25.0%)	0 (0.0%)	2 (66.7%)	18 (50.0%)	0.250
Attribution	Present	6 (31.6%)	5 (62.5%)	2 (50.0%)	0 (0.0%)	1 (33.3%)	14 (38.9%)	0.430
Disclosure	Present	3 (15.8%)	1 (12.5%)	0 (0.0%)	0 (0.0%)	0 (0.0%)	2 (5.6%)	0.816
Currency	Present	9 (47.4%)	6 (75.0%)	1 (25.0%)	1 (50.0%)	1 (33.3%)	18 (50.0%)	0.495
DISCERN rating	Poor	0 (0.0%)	0 (0.0%)	4 (100.0%)	2 (100.0%)	0 (0.0%)	6 (16.7%)	< 0.001^*^
	Moderate	19 (100.0%)	8 (100.0%)	0 (0.0%)	0 (0.0%)	3 (100.0%)	30 (83.3%)	
	High	0 (0.0%)	0 (0.0%)	0 (0.0%)	0 (0.0%)	0 (0.0%)	0 (0.0%)	

Med. Inst. & Hosp., Medical Institutions & Hospitals; GEW, General Educational Websites; DISCERN, Quality Assessment Instrument for Consumer Health Information, and JAMA; Journal of the American Medical Association Benchmark Criteria. ^*^p < 0.05.

Quality assessment using DISCERN showed that the mean total score was 39.72 ± 8.83, and no website achieved a high-quality rating (≥64). The majority of websites were rated as moderate quality (30/36, 83.3%), while the remainder were classified as poor. The quality of websites varied significantly across different affiliations. All medical institution/hospital websites and all health portals were rated as moderate quality according to DISCERN, while all governmental and public health organization websites were classified as poor quality (*p* < 0.001) (Table 1).

Readability analyses showed that the mean FKGL and SMOG scores were 3.65 ± 3.58 and 3.26 ± 0.79, respectively, with 69.4–97.2% of websites below a 7th-grade reading level, indicating easy-to-read content. FRE scores were very high (97.69 ± 6.39), with 97.2% of websites scoring ≥ 80, corresponding to “very easy” readability.

Correlation analysis demonstrated a significant positive association between JAMA and DISCERN scores (ρ = 0.430, *p* = 0.009). Strong negative correlations were observed between FRE and both FKGL (ρ = −0.704, *p* < 0.001) and SMOG (ρ = −0.684, *p* < 0.001). Weak, non-significant associations were observed between JAMA scores and all readability indices, as well as between DISCERN scores and readability indices (Table 2).

**Table 2:** Spearman's correlation of JAMA and DISCERN scores with readability indexes (*n* = 36).

**Variable**	**DISCERN**	**FRE**	**FKGL**	**SMOG**
JAMA score (total)	0.430^*^	−0.313	0.207	0.156
*p*-value	0.009	0.063	0.226	0.365
DISCERN score (total)	–	−0.118	0.031	0.277
*p*-value	–	0.492	0.856	0.102
FRE	–	–	−0.704^*^	−0.684^*^
*p*-value	–	–	0.000	0.000
FKGL	–	–	–	0.323
*p*-value	–	–	–	0.055

DISCERN, Quality Assessment Instrument for Consumer Health Information; JAMA, Journal of the American Medical Association Benchmark Criteria; FRE, Flesch Reading Ease; FKGL, Flesch-Kincaid Grade Level; SMOG, Simple Measure of Gobbledygook. ^*^p < 0.05.

## 4 Discussion

The internet serves as a key source for a wide range of health educational materials, presented in various formats, including auditory, visual, and written content. Although written materials are often considered an accessible way to acquire and understand information, concerns about their reliability remain widespread (7). This study represents the first comprehensive evaluation of Arabic-language online resources on IDA in terms of quality, readability, and reliability. Overall, while most websites were easy to read, they demonstrated only moderate quality and low reliability, with major insufficiencies in transparency, referencing, and coverage of treatment options, complications, and prevention.

The readability results from the current study indicate that most Arabic websites on IDA were written at a level appropriate for the general public, aligning with findings from previous evaluations of Arabic-language online health resources across various medical topics (18–21). In contrast, English-language IDA websites are typically written at higher grade levels, often exceeding recommended thresholds, which may reduce accessibility (22). However, the higher readability of Arabic content does not translate into higher quality. In fact, many websites failed to provide essential information such as treatment options, implications for quality of life, or consequences of untreated disease, consistent with findings from other Arabic-language health content studies, including breast cancer and oral health (23, 24). Major reliability concerns were also identified. Most resources failed to meet essential criteria such as addressing clinical uncertainty or providing adequate references, which are essential for informed decision-making. The attribution of sources and the disclosure of conflicts of interest were nearly absent, with only half of the resources indicating authorship and their currency. These findings suggest that simplification for general audiences does not guarantee comprehensive or reliable medical information. This may be attributed to several factors, including limited standardized editorial oversight or regulatory guidance for online health content in Arabic-speaking regions. In addition, some platforms may prioritize readability, user engagement, or commercial visibility over evidence-based accuracy, and in some cases, content creators may lack formal medical or health communication training, contributing to variability in accuracy and depth.

An important observation from this study is the general trend that higher-quality Arabic-language websites, as measured by DISCERN and JAMA scores, tended to be less readable, although correlations did not reach statistical significance. These findings suggest that websites of higher quality may use more advanced or complex language. This aligns with earlier research indicating that detailed, evidence-based health content often requires more complex text structures (18, 20). By contrast, English-language resources, while also sometimes difficult to read, more consistently achieve higher quality through transparent reporting of authorship, references, and update dates (22, 24). Moreover, quality also varied by website affiliation. Medical institutions, hospitals, and established health portals generally provided more reliable information. In contrast, governmental public health sites performed less consistently, possibly due to limited investment in digital patient education or an emphasis on brief awareness messaging rather than detailed guidance. Non-medical platforms, including blogs and commercial pharmacy websites, often prioritize accessibility or marketing visibility over evidence-based accuracy, and content is frequently produced by general writers rather than trained clinicians. As a result, information may be easy to read but lacks depth and proper sourcing, reinforcing that readability alone does not ensure trustworthy or comprehensive health information.

Findings from this study have practical and policy implications. Strengthening governance of online health information in Arabic-speaking regions, such as establishing national quality standards, mandatory source transparency, and promoting certification of trustworthy websites may help improve reliability of publicly accessible content. Additionally, encouraging healthcare institutions to produce accessible, evidence-based Arabic resources could further reduce reliance on commercial or informal platforms.

Ethically, the observed gaps in content are particularly concerning. While most Arabic websites provided basic information such as definitions, causes, and symptoms, essential details regarding treatment options, preventive strategies, and potential complications were frequently omitted. This pattern mirrors broader trends in Arabic-language digital health resources, where information is often simplified into brief lists rather than comprehensive clinical explanations (7, 19, 25). Easily accessible but low-quality information may mislead patients, weaken informed decision-making, and negatively influence health outcomes (7, 26). Such omissions can compromise patient autonomy and widen existing health disparities, particularly among individuals who rely on online sources as their main source of medical information.

This study has several notable strengths. It is among the first to systematically assess Arabic-language web resources on IDA using validated tools, ensuring objectivity and consistency in evaluating accuracy, clarity, and readability. It also highlights important gaps in content, particularly regarding prevention, complications, and treatment. Nonetheless, certain limitations should be acknowledged. Data were collected at a single time point, which may not capture subsequent updates. Only three major search engines were used. Additionally, the cross-sectional design precluded assessment of changes in quality over time. Future research should examine how variability in website authorship and editorial oversight affects content quality, and explore the role of multimedia and social media platforms in expanding the reach and impact of health education for Arabic-speaking populations.

In conclusion, while Arabic-language online resources for IDA are generally accessible in terms of readability, their quality and reliability remain suboptimal. This pronounced gap may contribute to misinformation, delayed care-seeking, and suboptimal management. Collaboration between medical institutions, public health organizations, and digital platforms will be essential for developing standardized, evidence-based patient education materials, which could support earlier intervention and help reduce the public health burden of IDA in Arabic-speaking communities.

## Data Availability

The original contributions presented in the study are included in the article/supplementary material, further inquiries can be directed to the corresponding author.
